# Supplementary material for: Shaping the quantum vacuum with anisotropic temporal boundaries
Source: Nanophotonics. 2022 Oct 19;12(3):539–48. doi: 10.1515/nanoph-2022-0491 (PMC11501409; doi:10.1515/nanoph-2022-0491)
Supplement: Supplementary file 1 — Supplementary Material Details [file j_nanoph-2022-0491_suppl.pdf]

Supplementary information for: Shaping the quantum vacuum with anisotropic temporal boundaries

J. Enrique Vázquez-Lozano[1] and Iñigo Liberal[1,*]

[1]*Department of Electrical, Electronic and Communications Engineering, Institute of Smart Cities (ISC), Public University of Navarre (UPNA), 31006 Pamplona, Spain*

I. REVIEW OF WAVE PROPAGATION AND QUANTIZATION IN ANISOTROPIC MEDIA

In this supplementary note we review the basics of wave propagation and the quantization of the electromagnetic field in anisotropic media, providing additional details to the theory developed in the main text.

A. Derivation of the wave equation and magnetic flux density

First, we derive the wave equation and a compact expression for the magnetic flux density. Our start point is time-harmonic Maxwell equations for plane wave modes with propagation constant $\mathbf{k} = \mathbf{s}\,k$:

$$\mathbf{k} \times \mathbf{E} = \omega \mathbf{B} \tag{1}$$

$$\mathbf{k} \times \mathbf{H} = -\omega \mathbf{D} \tag{2}$$

$$\mathbf{k} \cdot \mathbf{D} = 0 \tag{3}$$

$$\mathbf{k} \cdot \mathbf{B} = 0 \tag{4}$$

For an electrically isotropic medium ($\mathbf{D} = \varepsilon_0\,\varepsilon\,\mathbf{E}$) with anisotropic permeability ($\mathbf{B} = \mu_0\,\boldsymbol{\mu} \cdot \mathbf{H}$), we can derive the wave equation

$$\mathbf{k} \times \mathbf{k} \times \mathbf{H} = \mathbf{k} \cdot (\mathbf{k} \cdot \mathbf{H}) - k^2 \mathbf{H} = -\frac{\omega^2}{c^2}\,\varepsilon\,\boldsymbol{\mu} \cdot \mathbf{H} \tag{5}$$

With the definition $\eta = kc/\omega$, and combining both results we can rewrite the wave equation as

$$\mathbf{s}\,(\mathbf{s} \cdot \mathbf{H}) - \mathbf{H} = -\frac{1}{\eta^2}\varepsilon\,\boldsymbol{\mu} \cdot \mathbf{H} \tag{6}$$

This allow us to write a compact expression for the magnetic flux density:

$$\mathbf{B} = \mu_0 \boldsymbol{\mu} \cdot \mathbf{H} = \frac{\mu_0 \eta^2}{\varepsilon}\,(\mathbf{H} - \mathbf{s}\,(\mathbf{s} \cdot \mathbf{H})) \tag{7}$$

* Corresponding author: inigo.liberal@unavarra.es

B. Useful property for computing the energy of a mode

Next, we derive a useful property for determining the energy of a mode. First, we use (7) to write

$$\mathbf{H}^* \cdot \boldsymbol{\mu} \cdot \mathbf{H} = \frac{1}{\mu_0}\mathbf{H}^* \cdot \mathbf{B} = \frac{\eta^2}{\varepsilon}\left(\mathbf{H}^* \cdot \mathbf{H} - (\mathbf{s}\cdot\mathbf{H}^*)(\mathbf{s}\cdot\mathbf{H})\right) \tag{8}$$

Then, we use (2) to write

$$\mathbf{D}^* \cdot \mathbf{D} = \frac{\eta^2}{c^2}\left(\mathbf{s}\times\mathbf{H}^*\right)\cdot\left(\mathbf{s}\times\mathbf{H}\right) = \frac{\eta^2}{c^2}\left(\mathbf{H}^*\cdot\mathbf{H} - (\mathbf{s}\cdot\mathbf{H}^*)\cdot(\mathbf{s}\cdot\mathbf{H})\right) \tag{9}$$

Leading to the relation:

$$\mathbf{H}^* \cdot \boldsymbol{\mu} \cdot \mathbf{H} = \frac{c^2}{\varepsilon}\mathbf{D}^* \cdot \mathbf{D} \tag{10}$$

Finally, if we define $\mathbf{H} = \mathbf{h_k} H_0$, then we can compactly write $\mathbf{D} = -\frac{k}{\omega}\mathbf{s}\times\mathbf{h_k}H_0$ and

$$\mathbf{h_k}\cdot\boldsymbol{\mu}\cdot\mathbf{h_k} = \frac{k^2c^2}{\omega^2\varepsilon}\left(\mathbf{s}\times\mathbf{h_k}\right)^2 \tag{11}$$

C. Review of the quantization procedure for anisotropic dielectrics

Next, we carry out the quantization of the plane-wave modes discussed above

$$\mathbf{H_k}(\mathbf{r},t) = P_\mathbf{k}\,\frac{1}{\sqrt{V}}\,\mathbf{h_k}\,\alpha_\mathbf{k}(t)\,e^{i\mathbf{k}\cdot\mathbf{r}} \tag{12}$$

$$\mathbf{E_k}(\mathbf{r},t) = -\frac{1}{\omega\varepsilon_0\varepsilon}\mathbf{k}\times\mathbf{H_k}(\mathbf{r},t) = -P_\mathbf{k}\frac{1}{\sqrt{V}}\frac{\mathbf{k}}{\omega\varepsilon_0\varepsilon}\left(\mathbf{u_k}\times\mathbf{h_k}\right)\alpha_\mathbf{k}(t)\,e^{i\mathbf{k}\cdot\mathbf{r}} \tag{13}$$

where we have introduced pre-factors in the form of a quantization volume V, a normalization constant $P_\mathbf{k}$ and a dynamical variable $\alpha_\mathbf{k}(t)$. The energy density is given by

$$\mathcal{U}_\mathbf{k}(t) = \int_V d^3\mathbf{r}\,\left(\varepsilon_0\,\varepsilon\,\left|\mathbf{E_k}(\mathbf{r},t)\right|^2 + \mu_0\,\mathbf{H}^*_\mathbf{k}(\mathbf{r},t)\cdot\boldsymbol{\mu}\cdot\mathbf{H_k}(\mathbf{r},t)\right) \tag{14}$$

$$= \frac{\mu_0\,P_\mathbf{k}^2}{2}\left(\alpha^*_\mathbf{k}(t)\,\alpha_\mathbf{k}(t) + \alpha_\mathbf{k}(t)\,\alpha^*_\mathbf{k}(t)\right)\left(\frac{k^2c^2}{\omega^2\varepsilon}\left(\mathbf{u_k}\times\mathbf{h}_k\right)^2 + \mathbf{h}_k\cdot\boldsymbol{\mu}\cdot\mathbf{h}_k\right) \tag{15}$$

Using the property (11) the energy density can be compactly written as

$$\mathcal{U}_\mathbf{k}(t) = \mu_0\,P_\mathbf{k}^2\,\mathbf{h_k}\cdot\boldsymbol{\mu}\cdot\mathbf{h_k}\,\left(\alpha^*_\mathbf{k}(t)\,\alpha_\mathbf{k}(t) + \alpha_\mathbf{k}(t)\,\alpha^*_\mathbf{k}(t)\right) \tag{16}$$

Quantization is carried out by using the substitution $\alpha_\mathbf{k} \to \sqrt{\hbar\omega_\mathbf{k}/2}\,\widehat{a}_\mathbf{k}$:

$$\widehat{\mathcal{H}}_\mathbf{k} = P_\mathbf{k}^2\;\mathbf{h_k}\cdot\boldsymbol{\mu}\cdot\mathbf{h_k}\;\mu_0\,\frac{\hbar\omega_\mathbf{k}}{2}\,\left(\widehat{a}^\dagger_\mathbf{k}\widehat{a}_\mathbf{k} + \widehat{a}_\mathbf{k}\widehat{a}^\dagger_\mathbf{k}\right) \tag{17}$$

And we choose the normalization constant such that

$$P_{\mathbf{k}} = \sqrt{\frac{1}{\mu_0\, C_{\mathbf{k}}}} \tag{18}$$

with

$$C_{\mathbf{k}} = \mathbf{h}_{\mathbf{k}} \cdot \boldsymbol{\mu} \cdot \mathbf{h}_{\mathbf{k}} \tag{19}$$

obtaining the usual expression for the Hamiltonian

$$\widehat{\mathcal{H}}_{\mathbf{k}} = \frac{\hbar\omega_{\mathbf{k}}}{2} \left(\widehat{a}_{\mathbf{k}}^{\dagger}\widehat{a}_{\mathbf{k}} + \widehat{a}_{\mathbf{k}}\widehat{a}_{\mathbf{k}}^{\dagger}\right) \tag{20}$$

Then, the (positive frequency) field operators are given by

$$\widehat{\mathbf{H}}_{\mathbf{k}}^{(+)}(\mathbf{r}) = \sqrt{\frac{\hbar\omega_{\mathbf{k}}}{2\mu_0 C_{\mathbf{k}} V}}\; \mathbf{h}_{\mathbf{k}}\; \widehat{a}_{\mathbf{k}}\; e^{i\mathbf{k}\cdot\mathbf{r}} \tag{21}$$

and the complete operators are given by $\widehat{\mathbf{H}}_{\mathbf{k}}(\mathbf{r}) = \widehat{\mathbf{H}}_{\mathbf{k}}^{(+)}(\mathbf{r}) + h.c.$.

D. Input-output relations for an anisotropic temporal boundary

Here we provide additional details for the derivation of Eq. (2) of the main text, describing the input-output relations for an anisotropic temporal boundary. First, following the expression for the magnetic field operator we can construct the magnetic flux $\widehat{\mathbf{B}}_{\mathbf{k}}(\mathbf{r})$ and the electric displacement operators as follows

$$\widehat{\mathbf{B}}_{\mathbf{k}}(\mathbf{r}) = \mu_0 \boldsymbol{\mu} \cdot \mathbf{h}_{\mathbf{k}} \sqrt{\frac{\hbar\omega_{\mathbf{k}}}{2\mu_0 C_{\mathbf{k}} V}} \left(\widehat{a}_{\mathbf{k}} e^{i\mathbf{k}\cdot\mathbf{r}} + \widehat{a}_{\mathbf{k}}^{\dagger} e^{-i\mathbf{k}\cdot\mathbf{r}}\right) \tag{22}$$

$$\widehat{\mathbf{D}}_{\mathbf{k}}(\mathbf{r}) = -\frac{k}{\omega_{\mathbf{k}}} \sqrt{\frac{\hbar\omega_{\mathbf{k}}}{2\mu_0 C_{\mathbf{k}} V}} \left(\mathbf{u}_{\mathbf{k}} \times \mathbf{h}_{\mathbf{k}}\right) \left(\widehat{a}_{\mathbf{k}} e^{i\mathbf{k}\cdot\mathbf{r}} - \widehat{a}_{\mathbf{k}}^{\dagger} e^{-i\mathbf{k}\cdot\mathbf{r}}\right) \tag{23}$$

Then, recalling the property (11), we can multiply (22) by $\mathbf{h}_{\mathbf{k}}$ and find that the continuity of $\widehat{\mathbf{B}}_{k}(\mathbf{r})$ leads to the condition

$$\sqrt{\omega_{\mathbf{k}1}\varepsilon_1} \left(\widehat{a}_{\mathbf{k}1} + \widehat{a}_{-\mathbf{k}1}^{\dagger}\right) = \sqrt{\omega_{\mathbf{k}2}\varepsilon_2} \left(\widehat{a}_{\mathbf{k}2} + \widehat{a}_{-\mathbf{k}2}^{\dagger}\right) \tag{24}$$

Similarly, we can multiply (23) by $\mathbf{u}_{\mathbf{k}} \times \mathbf{h}_{\mathbf{k}}$ and obtain the condition

$$\sqrt{\frac{1}{\omega_{\mathbf{k}1}\varepsilon_1}} \left(\widehat{a}_{\mathbf{k}1} - \widehat{a}_{-\mathbf{k}1}^{\dagger}\right) = \sqrt{\frac{1}{\omega_{\mathbf{k}2}\varepsilon_2}} \left(\widehat{a}_{\mathbf{k}2} - \widehat{a}_{-\mathbf{k}2}^{\dagger}\right) \tag{25}$$

Finally, solving (24)-(25) leads to the input-output relations as given by Eq. (2) of the main text.

E. Particularization to a diagonal permeability tensor

Let us assume that we have a diagonal permeability tensor: $\boldsymbol{\mu} = \mathrm{diag}\left\{\mu_x, \mu_y, \mu_z\right\}$. Then, each component of Eq. (7) can be rearranged as follows

$$H_i = \frac{\eta^2}{\eta^2 - \varepsilon\mu_i}\, s_i\left(\mathbf{s}\cdot\mathbf{H}\right) \quad i = x, y, z \tag{26}$$

Multiplying on both sides by s_i and summing over i we get

$$\mathbf{s}\cdot\mathbf{H} = \sum_i \frac{\eta^2}{\eta^2 - \varepsilon\mu_i}\, s_i^2\left(\mathbf{s}\cdot\mathbf{H}\right) \tag{27}$$

Noting that $\mathbf{s}\cdot\mathbf{H}$ is on both sides of the equation and it can be removed, and dividing by η^2, we obtain the dispersion relation

$$\frac{s_x^2}{\eta^2 - \varepsilon\mu_x} + \frac{s_y^2}{\eta^2 - \varepsilon\mu_y} + \frac{s_z^2}{\eta^2 - \varepsilon\mu_z} = \frac{1}{\eta^2} \tag{28}$$

For 2D propagation $\mathbf{s} = \mathbf{u}_x s_x + \mathbf{u}_y s_y = \mathbf{u}_x\mathrm{cos}\phi + \mathbf{u}_y\mathrm{sin}\phi$, so that we can write

$$\frac{s_x^2}{\eta^2 - \varepsilon\mu_x} + \frac{s_y^2}{\eta^2 - \varepsilon\mu_y} = \frac{1}{\eta^2}$$

$$\eta^2\left(s_x^2\left(\eta^2 - \varepsilon\mu_y\right) + s_y^2\left(\eta^2 - \varepsilon\mu_x\right)\right) = \left(\eta^2 - \varepsilon\mu_x\right)\left(\eta^2 - \varepsilon\mu_y\right)$$

$$-\eta^2\varepsilon\left(s_x^2\mu_y + \eta^2 s_y^2\mu_x\right) = -\eta^2\varepsilon\left(\mu_x + \mu_y\right) + \varepsilon^2\mu_x\mu_y$$

$$\eta^2\left(\mu_x + \mu_y - s_x^2\mu_y - s_y^2\mu_x\right) = \varepsilon\mu_x\mu_y$$

$$\eta^2\left(\mu_x s_x^2 + \mu_y s_y^2\right) = \varepsilon\mu_x\mu_y$$

$$\frac{s_x^2}{\mu_y} + \frac{s_y^2}{\mu_x} = \frac{\varepsilon}{\eta^2}$$

and the dispersion relation reduces to

$$\frac{\cos^2\phi}{\mu_y} + \frac{\sin^2\phi}{\mu_x} = \frac{\varepsilon}{\eta^2} \tag{29}$$

In this manner, the effective refractive index, the wavenumber and the frequency are given by

$$\eta = \sqrt{\frac{\varepsilon \mu_x \mu_y}{\mu_x \cos^2\phi + \mu_y \sin^2\phi}} \tag{30}$$

$$\omega_{\mathbf{k}} = kc\sqrt{\frac{\mu_x \cos^2\phi + \mu_y \sin^2\phi}{\varepsilon \mu_x \mu_y}} \tag{31}$$
